# Functional Characterization of *AmGPPS/GGPPS* Gene Family in *Antirrhinum majus* and the Regulatory Role of *AmGPPS6* in Floral Scent Variation

**DOI:** 10.3390/plants15101457

**Published:** 2026-05-10

**Authors:** Shaorong Dong, Banghan Liu, Jiongli Chen, Chong Ma, Shuangshuang Cao, Haoyue Wang, Senbao Shi, Xiaohui Song, Longqing Chen, Zhenglin Qiao

**Affiliations:** 1 Yunnan Province Engineering Research Center for Functional Flower Resources and Industrialization, College of Landscape Architecture and Horticulture, Southwest Forestry University, Kunming 650224, China; dongsr@swfu.edu.cn (S.D.); liubh@swfu.edu.cn (B.L.); chenjl@swfu.edu.cn (J.C.); machong@swfu.edu.cn (C.M.); caoss@swfu.edu.cn (S.C.); wanghy@swfu.edu.cn (H.W.); shisb@swfu.edu.cn (S.S.); songxh@swfu.edu.cn (X.S.); 2 Yunnan Key Laboratory of Landscape Plant Resource Cultivation and Application, Yunnan Academy of Forestry and Grassland Sciences, Kunming 650224, China

**Keywords:** *Antirrhinum majus*, floral scent, geranyl diphosphate synthase (GPPS), GPPS small subunit, gene family, functional validation

## Abstract

Geranyl diphosphate synthase (GPPS) is a key enzyme in the plant isoprenoid metabolic pathway and regulates the biosynthesis of volatile monoterpenes. It plays an important role in the biosynthesis of floral volatile terpenoids (FVTs) and inter-cultivar variation in snapdragon. Despite its importance in floral scent formation, the *GPPS/GGPPS* gene family in snapdragon (*Antirrhinum majus* L.) has not been systematically characterized. In this study, nine GPPS/GGPPS family members were identified at genome-wide level. These include six *AmGPPS* and three *AmGGPPS* genes. Phylogenetic analysis grouped them into distinct subfamilies. We further analyzed their chromosomal locations, gene structures, conserved protein motifs, and promoter cis-acting elements. These results revealed both conservation and functional divergence within the gene family. To explore their functional roles, we compared gene expression profiles at the full flowering stage. This comparison was performed between strongly scented cultivar (Am3) and the weakly scented cultivar (Am5). Among all candidates, *AmGPPS6* showed the most significant differential expression. Further, functional validation was conducted using transient overexpression and virus-induced gene silencing (VIGS). Overexpression of *AmGPPS6* significantly increased terpenoid production. Total floral volatile terpenoids (FVTs) increased by 1.4 fold. Both monoterpene and sesquiterpene emissions were enhanced. In contrast, silencing of *AmGPPS6* markedly reduced the emission of key monoterpenes such as ocimene and its isomers. Sequence analysis showed that *AmGPPS6* shares 67.04% identity with canonical GPPS small subunit (GPPS.SSU). However, it lacks the conserved catalytic DDx2-D motif. This suggests that *AmGGPPS2* is not catalytically active. Instead, it likely functions through heterodimer with *AmGGPPS2*. This interaction is supported by coordinated transcriptional expression patterns. Additionally, natural sequence polymorphisms were identified in GPPS.SSU. These variations, rather than those in GPPS.LSU, appear to drive differences in monoterpense emission between cultivars. In conclusion, *AmGPPS6* in a key regulator of floral scent biosynthesis in snapdragon. This study provides new insights into functional roles of *GPPS/GGPPS* genes. It also offers valuable gene targets for the molecular breeding of aromatic traits in ornamental plants.

## 1. Introduction

Terpenoids represent the most structurally and functionally diverse class of plant specialized metabolites, with over 55,000 known compounds documented to date [1]. Among these, floral volatile terpenoids (FVTs) play essential ecological roles. They attract pollinators, mediate defense against pathogens and herbivores, and recruit natural enemies, thereby substantially enhancing plant fitness and reproductive success in complex environments [2,3]. Monoterpenes constitute core components of floral scent profiles and are critical for attracting specialized pollinators, ensuring successful plant reproduction [4]. The biosynthesis of FVTs is primarily dependent on two compartmentalized pathways: the cytosolic mevalonate (MVA) pathway and the plastidial 2-C-methyl-D-erythritol-4-phosphate (MEP) pathway. These pathways generate the universal five-carbon building blocks isopentenyl diphosphate (IPP) and its allylic isomer dimethylallyl diphosphate (DMAPP) [4,5,6]. The biosynthesis of volatile monoterpenes specifically relies on the condensation of IPP and DMAPP within plastids to produce geranyl diphosphate (GPP, C10) and geranylgeranyl diphosphate (GGPP, C20) [4,6]. These condensation reactions are catalyzed by geranyl diphosphate synthase (GPPS) and geranylgeranyl diphosphate synthase (GGPPS), respectively. Subsequently, monoterpene synthases (monoTPSs) utilize GPP as a substrate to generate a diverse array of monoterpenes [7].

The *GPPS* gene has been extensively investigated across angiosperms. Sweet pepper (*Capsicum annuum*) was among the first angiosperm species from which the GPPS gene was cloned, establishing a foundation for subsequent research [8,9]. Building upon this, studies on the GPPS small subunit (GPPS.SSU) have been conducted in diverse plant species, primarily focusing on its regulatory mechanism underlying volatile monoterpene biosynthesis. In model plants such as *Arabidopsis thaliana* [10] and *Oryza sativa* [11], research has elucidated the canonical paradigm wherein the small subunit heterodimerizes with the large subunit (GPPS.LSU) to modulate GPP synthesis [10]. In fragrant flowers and ornamental plants, including snapdragon (*Antirrhinum majus*) [12,13], peppermint (*Mentha x piperita*) [14,15,16], gardenia (*Gardenia jasminoides*) [17], and wintersweet (*Chimonanthus praecox*) [18], investigations have centered on the pivotal role of the small subunit in floral scent production. Collectively, these studies have established that angiosperm GPPS predominantly functions as a heterodimer, comprising a non-catalytic small subunit (GPPS.SSU) and a catalytic large subunit (GPPS.LSU) [19,20]. The small subunit regulates GPP synthesis through heterodimer formation with the large subunit, representing a conserved mechanism influencing plant aroma and specialized metabolite biosynthesis [19,20,21].

Further investigations have revealed that homodimers of GPPS.LSU exhibit GGPPS activity, catalyzing GGPP synthesis [19,22]. However, when the large subunit interacts with the small subunit (particularly SSU I) to form a heterodimer, the enzyme’s activity shifts toward catalyzing GPP production [13,19,20]. This mechanistic switch is highly conserved in monoterpene biosynthesis among angiosperms, as demonstrated in species such as rose [23], lily (*Lilium* spp.), snapdragon, tobacco [24], and sweet osmanthus (*Osmanthus fragrans*) [23,24,25]. Notably, angiosperms possess multiple GGPPS/GPPS.LSU proteins that, through specific interactions with the small subunit, precisely orchestrate the metabolic partitioning between GPP and GGPP, thereby influencing the biosynthesis of distinct downstream terpenoid classes [19,20,25]. Phylogenetically, GPPS.SSU can be classified into two clades: SSU I and SSU II. SSU II contains a DD(X)_2–4_D motif enabling substrate binding, whereas SSU I lacks this motif and is therefore catalytically inactive on its own, requiring interaction with GGPPS/GPPS.LSU proteins—which possess two DD(X)_2–4_D domains—to fulfill its function [26,27]. Although GGPPS/GPPS.LSU genes are expressed across a broad range of organs, including leaves and flowers, GPPS.SSU expression is intimately associated with monoterpene biosynthesis in angiosperms [19,20,23,28].

Currently, the *GPPS/GGPPS* gene family in snapdragon has not been systematically characterized, leaving the functional differentiation and expression patterns among family members largely unclear. Furthermore, existing functional studies have predominantly relied on heterologous systems, lacking direct in planta validation within homologous contexts [13]. In this study, we employed two snapdragon cultivars exhibiting distinct floral scent intensities—the strongly scented Am3 and the weakly scented Am5—to systematically identify *GPPS/GGPPS* gene family members at the genome-wide level. Through integration with expression profiling during the full flowering stage, *AmGPPS6* was identified as a candidate gene potentially underlying differential monoterpene emission. Functional validation via transient overexpression and virus-induced gene silencing (VIGS) in homologous in planta systems revealed that the expression level of *AmGPPS6* exhibits a significant positive correlation with monoterpene emission. The protein encoded by *AmGPPS6* shares sequence homology with canonical GPPS small subunits, and alterations in its expression concurrently affect the transcript levels of *AmGGPPS2*, suggesting that these two proteins may cooperatively regulate monoterpene biosynthesis through heterodimer formation. These results demonstrate that *AmGPPS6* is the key gene responsible for inter-cultivar variation in monoterpene emission in snapdragon. Our findings elucidate the molecular basis of floral scent diversity from the perspective of substrate supply, refine our understanding of the functional configuration of GPPS small subunits within snapdragon cultivars, and provide both a theoretical foundation and candidate genetic targets for molecular breeding of aromatic traits in ornamental plants.

## 2. Results

### 2.1. Identification and Physicochemical Characterization of AmGPPS/AmGGPPS Gene Family Members in Snapdragon

To comprehensively characterize the fundamental features of the *AmGPPS/AmGGPPS* gene families in snapdragon, a total of nine genes were identified in this study. Based on their chromosomal distribution, these were designated as *AmGPPS1* to *AmGPPS6* and *AmGGPPS1* to *AmGGPPS3*. Systematic identification and systematic identification and analysis of physicochemical properties of the AmGPPS/AmGGPPS proteins were subsequently performed. As summarized in Table 1, the proteins encoded by these nine genes range in length from 297 to 416 amino acids, with molecular weights spanning 32.43 to 46.30 kDa and theoretical isoelectric points varying between 5.48 and 7.73. With the exception of *AmGPPS3*, *AmGGPPS2*, and *AmGGPPS3*, all proteins are predicted to be stable. All family members exhibit predominantly hydrophilic characteristics. Subcellular localization predictions indicate that these proteins are localized to both the cytoplasm and chloroplasts.

### 2.2. Chromosomal Localization of AmGPPS/AmGGPPS Gene Family Members in Snapdragon

**Figure 1:**
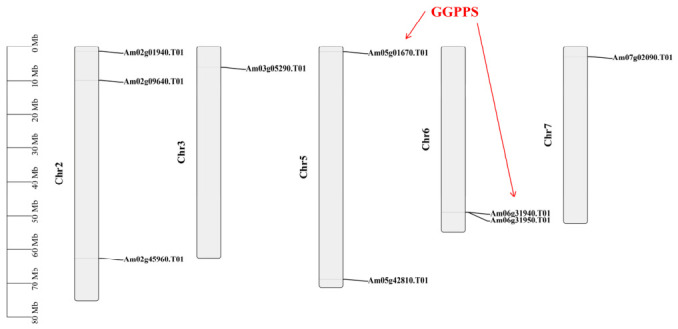
Chromosomal localization of the *AmGPPS*/*AmGGPPS* gene family members. The physical positions of nine *AmGPPS/AmGGPPS* genes on the chromosomes of *A. majus* are shown. Chromosome numbers (Chr2–Chr7) are labeled below each chromosome bar, and the scale bar on the left indicates chromosome length in megabase pairs (Mb). The gene family members are unevenly distributed across five chromosomes: three members (*Am02g01940.T01*, *Am02g09640.T01*, *Am02g45960.T01*) are located on Chr2, one (*Am03g05290.T01*) on Chr3, two (*Am05g01670.T01*, *Am05g42810.T01*) on Chr5, two (*Am06g31940.T01*, *Am06g31950.T01*) on Chr6, and one (*Am07g02090.T01*) on Chr7. Red arrows highlight the *AmGGPPS* genes, including *Am05g01670.T01* and the tandemly duplicated gene pair *Am06g31940.T01* and *Am06g31950.T01* on Chr6.

To elucidate the genomic distribution of the *AmGPPS*/*AmGGPPS* gene family, we further analyzed their chromosomal localization. Based on validation using the Pfam_scan and SMART databases, the nine identified members are unevenly distributed across chromosomes 2, 3, 5, 6, and 7 (Figure 1). Chromosome 2 exhibits the highest concentration, harboring three genes, a pattern that may be related to chromosome size.

### 2.3. Analysis of Gene Structure, Protein Characteristics, and Promoters of the AmGPPS/AmGGPPS Gene Family in Snapdragon

#### 2.3.1. Analysis of Gene Structure and Conserved Protein Motifs

**Figure 2:**
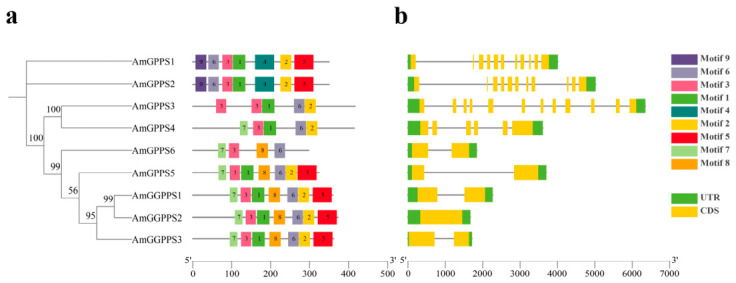
Analysis of *AmGPPS*/*AmGGPPS* Gene Structure and Conserved Protein Motifs. (**a**) The unrooted neighbor-joining phylogenetic tree of *AmGPPS/AmGGPPS* proteins is shown on the left, with bootstrap values indicated at the nodes. The distribution of nine conserved motifs (Motif 1–9) identified by MEME analysis is displayed on the right. Each colored box represents a specific motif, and the scale at the bottom indicates the position of the motifs in amino acid residues. (**b**) Exon-intron organization of *AmGPPS/AmGGPPS* genes. Green boxes represent 5′ and 3′ untranslated regions (UTRs), yellow boxes represent exons (coding sequences, CDS), and black lines represent introns. The scale bar at the bottom indicates the length of the gene sequence in base pairs (bp). Members within the same phylogenetic clade show similar motif compositions and gene structures, indicating functional conservation within subgroups.

To understand the structural diversity of *AmGPPS*/*AmGGPPS* genes and the functional domain composition of their encoded proteins, we analyzed the gene structures and conserved protein motifs of the nine *AmGPPS*/*AmGGPPS* genes in snapdragon. The results indicate that *AmGPPS1* and *AmGPPS2* share similar exon-intron structures with identical exon counts (Figure 2b). *AmGPPS3* contains the highest number of exons and introns, comprising 12 exons and 11 introns. *AmGPPS4* exhibits a distinct exon-intron structure and count compared to other members. *AmGPPS5*, *AmGPPS6*, *AmGGPPS1*, and *AmGGPPS3* possess similar exon-intron structures and counts. Notably, *AmGGPPS2* lacks introns.

Analysis of protein motifs (Figure 2a) provides insights into the potential functions of AmGPPS/AmGGPPS proteins. The analysis revealed that all nine genes contain Motifs 3 and 6. With the exception of *AmGPPS6*, the remaining eight members contain Motifs 1 and 2. Furthermore, identical motif compositions were observed between *AmGPPS1* and *AmGPPS2*, as well as between *AmGPPS5* and *AmGGPPS*.

#### 2.3.2. Multiple Sequence Alignment and Phylogenetic Analysis

**Figure 3:**
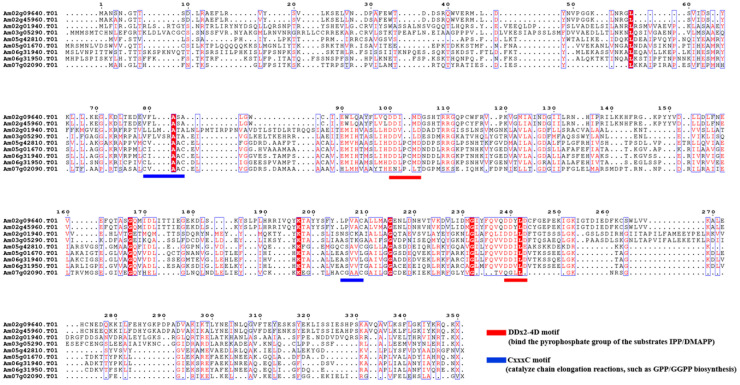
Multiple sequence alignment of the AmGPPS/AmGGPPS protein. Amino acid sequences of the *AmGPPS/AmGGPPS* family members were aligned to identify conserved domains and functional motifs. Conserved residues are highlighted in blue boxes, with identical amino acids shaded in red. The two key functional motifs are annotated: the DDx_2–4_D motif (red bars), which binds the pyrophosphate moiety of the substrates isopentenyl diphosphate (IPP) and dimethylallyl diphosphate (DMAPP); and the CxxxC motif (blue bars), which is essential for catalyzing the chain elongation reactions in GPP/GGPP biosynthesis.

To elucidate the evolutionary relationships and functional differentiation of AmGPPS/AmGGPPS proteins in snapdragon relative to orthologs from other species, a phylogenetic tree was constructed and multiple sequence alignments were performed in this study (Figure 3). Using the Neighbor-Joining method, a phylogenetic tree was generated comprising the nine identified AmGPPS/AmGGPPS proteins together with functionally characterized GPPS/GGPPS proteins from diverse species. The resulting topology revealed that six proteins clustered within the *GPPS* family, while three proteins grouped within the *GGPPS* family. Notably, *AmGPPS6* exhibited the closest phylogenetic relationship with MpGPPS.SSU from peppermint (*M. piperita*, *AAF08792.1*) and demonstrated exceptionally high sequence homology with AmGPPS.SSU, sharing 67.04% sequence identity.

Multiple sequence alignment of protein sequences revealed that all *AmGGPPS* members contain one “CxxxC” motif and two “DDx_2–4_D” motifs. Among the *AmGPPS* members, *AmGPPS1*, *AmGPPS2*, *AmGPPS3*, and *AmGPPS4* each possess two “DDx_2–4_D” motifs; *AmGPPS5* contains two “CxxxC” motifs and one “DDx_2–4_D” motif; and *AmGPPS6* harbors two “CxxxC” motifs. Functionally, the “CxxxC” motif serves as an auxiliary element contributing to structural stability and activity regulation, whereas the “DDx_2–4_D” motif constitutes the core domain responsible for substrate binding and catalytic activity. Collectively, this motif architecture indicates that the *AmGGPPS* and *AmGPPS* subfamilies maintain relatively conserved domain structures, suggesting their functional specialization has been preserved throughout evolutionary diversification.

#### 2.3.3. Analysis of Cis-Acting Elements in Promoters

**Figure 4:**
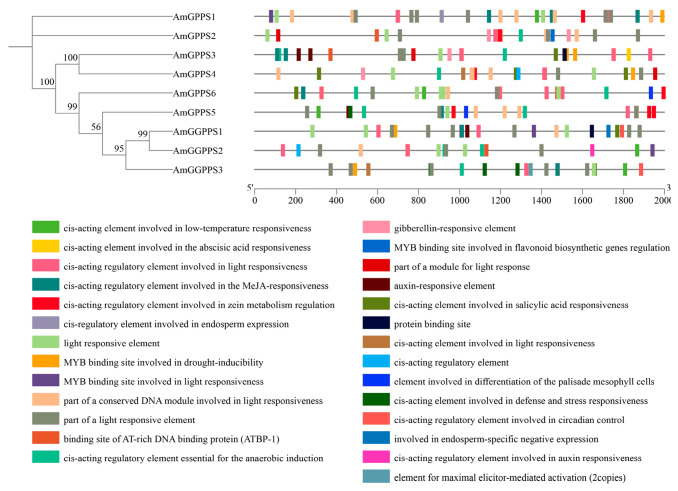
Analysis of cis-acting elements in the promoters of *AmGPPS/AmGGPPS* genes in Snapdragon. Phylogenetic relationships among *AmGPPS/AmGGPPS* genes are presented on the left, with bootstrap support values indicated at key nodes. The schematic on the right illustrates the distribution of diverse cis-acting regulatory elements identified in the 2000-bp upstream promoter sequences. Each colored box represents a specific element, with functions categorized in the legend: light responsiveness, hormone signaling (gibberellin, abscisic acid, methyl jasmonate, auxin, salicylic acid), abiotic stress responses (low temperature, drought, anaerobic induction), and developmental regulation (endosperm expression, circadian control).

To investigate the potential regulatory mechanisms governing *AmGPPS/AmGGPPS* gene expression, the cis-acting regulatory elements within their promoter regions were analyzed (Figure 4). These cis-elements were categorized into five major classes based on their predicted functions: light-responsive elements, elements associated with environmental stress responses, elements involved in phytohormone signaling, elements related to plant growth and development, and other regulatory elements. Statistical analysis revealed that among the promoter elements identified in the *AmGPPS/AmGGPPS* gene family, light-responsive elements constituted the most abundant category, whereas growth and development-related elements were the least represented.

#### 2.3.4. Synteny Analysis

**Figure 5:**
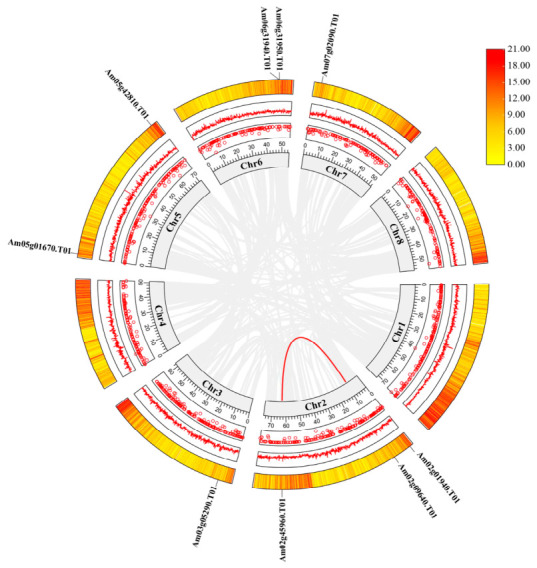
Intraspecific collinearity analysis of *AmGPPS/AmGGPPS* genes in Snapdragon. Circular diagram showing collinearity among *AmGPPS/AmGGPPS* genes in the snapdragon genome. Chromosomes (Chr1–Chr8) are arranged in a circle, with gene names labeled on the periphery. The heatmap on each chromosome represents the gene density (color scale on the right, 0–21.00).

**Figure 6:**
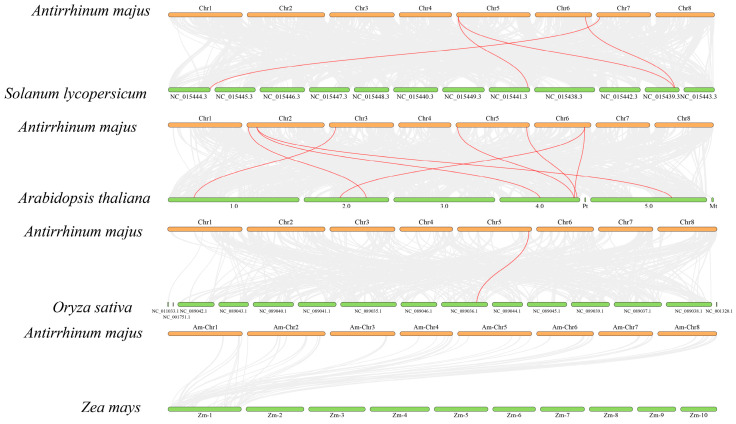
Inter-species collinearity analysis of *AmGPPS*/*AmGGPPS* genes. Collinearity of *AmGPPS/AmGGPPS* genes between *A. majus* and four other plant species (S. *lycopersicum*, *A. thaliana*, *O. sativa*, and *Zea mays*) is illustrated. Chromosomes are displayed as horizontal bars, with *A. majus* chromosomes (Chr1–Chr8) aligned with those of the other species. Gray lines in the background represent all syntenic blocks between the genomes, while red lines specifically highlight the collinear orthologous pairs of *AmGPPS/AmGGPPS* genes.

To investigate the conservation and diversification of *AmGPPS/AmGGPPS* genes from an evolutionary genomic perspective, both intra- (Figure 5) and inter-species (Figure 6) synteny analyses were conducted. Intra-species synteny analysis revealed that *AmGPPS1* (*Am02g09640.T01*) and *AmGPPS2* (*Am02g45960.T01*) are paralogous genes, both localized on chromosome 2 (Figure 1), suggesting that these two genes originated from a tandem duplication event. The remaining seven genes exhibited no detectable intra-species syntenic signals, indicating the absence of clustered gene duplications and lacking spatial association with duplication events within the genome.

Inter-species synteny analysis of *AmGPPS/GGPPS* genes (Figure 6; Table 2) demonstrated that snapdragon, a dicotyledonous plant, shares four syntenic gene pairs with tomato (*Solanum lycopersicum*) and eight syntenic gene pairs with *A. thaliana*—both also dicotyledons—yielding a total of nine orthologous genes identified across these comparisons. In contrast, only one syntenic gene pair and four orthologous genes were identified between snapdragon and rice (*O. sativa*), a monocotyledonous species. This pronounced disparity not only reflects the evolutionary conservation of the *GPPS/GGPPS* gene family within dicotyledons, confirming that snapdragon exhibits typical dicotyledonous characteristics with respect to this gene family, but also provides an evolutionary framework for inferring the biological functions of *AmGPPS/GGPPS* family members based on orthologous gene functionality in related species.

### 2.4. Expression Patterns of AmGPPS and AmGGPPS Gene Family Members

**Figure 7:**
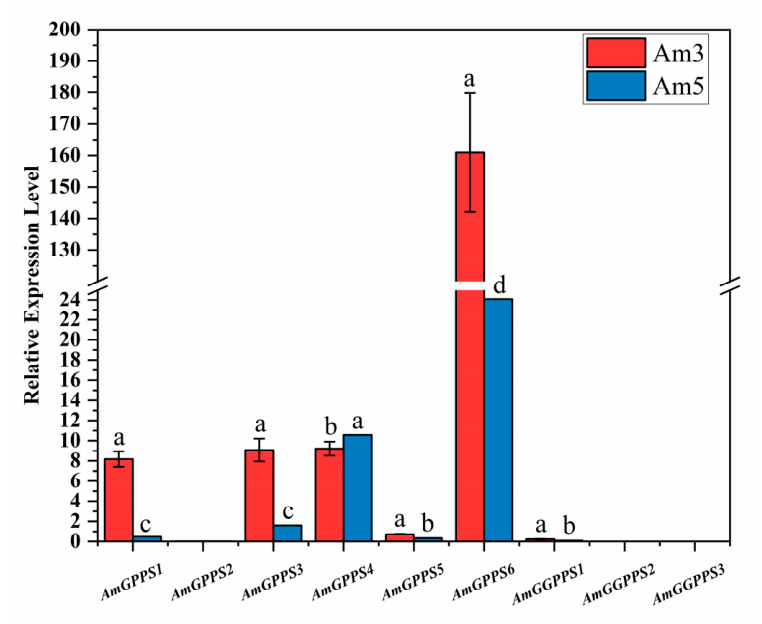
Expression patterns of the *AmGPPS* and *AmGGPPS* gene family members. All data are presented as mean ± SD (n = 3 biological replicates). Different lowercase letters above the bars indicate statistically significant differences (*p* < 0.05, one-way ANOVA followed by Tukey’s multiple range test). Note: the *Y*-axis is broken to accommodate the large expression range of *AmGPPS6*.

Based on the aforementioned bioinformatic characterization of the *AmGPPS/AmGGPPS* gene family, we sought to identify candidate genes most closely associated with floral scent emission in snapdragon. To this end, the expression profiles of all nine family members were comparatively analyzed in petals at the full flowering stage between two cultivars exhibiting differential scent emission: the strongly scented Am3 and the weakly scented Am5 (Figure 7).

The results revealed that six genes were differentially expressed between Am3 and Am5. Notably, *AmGPPS2*, *AmGGPPS2*, and *AmGGPPS3* exhibited extremely low transcript abundances in both cultivars. Among the differentially expressed genes, *AmGPPS6* displayed not only relatively high expression levels overall but also the most pronounced inter-cultivar difference, with transcript accumulation being significantly higher in the strongly scented Am3 compared to the weakly scented Am5. In contrast, the remaining differentially expressed genes, while showing significant variation between cultivars, were expressed at considerably lower levels. Given its prominent expression pattern tightly correlated with the floral scent phenotype, *AmGPPS6* was selected as the primary candidate for subsequent functional characterization. These results indicated that *AmGPPS6* exhibited the most significant expression difference between Am3 and Am5 and was strongly associated with scent intensity. However, floral scent variation is a complex metabolic trait, which may be coordinatively determined by upstream precursor supply genes, downstream terpene synthase genes, and transcription factors.

### 2.5. Preliminary Functional Characterization of the AmGPPS6 Gene

To elucidate the biological function of *AmGPPS6* in floral scent biosynthesis in snapdragon, functional characterization was performed using transient overexpression and VIGS approaches. Using the pCAMBIA1300-35S-E9 (Bio-Transduction Lab, Wuhan, China) and pTRV2 (Bio-Transduction Lab, Wuhan) vectors as positive controls, the recombinant constructs pCAMBIA1300-35S-E9-*AmGPPS6* and pTRV2-*AmGPPS6* were introduced into inflorescences of cut snapdragon flowers via vacuum infiltration. Specifically, the overexpression construct was infiltrated into the weakly scented cultivar Am5, while the VIGS construct was infiltrated into the strongly scented cultivar Am3, enabling preliminary investigation of *AmGPPS6* function through gain- and loss-of-function strategies, respectively.

#### 2.5.1. Transient Overexpression of the AmGPPS6 Gene in Snapdragon

**Figure 8:**
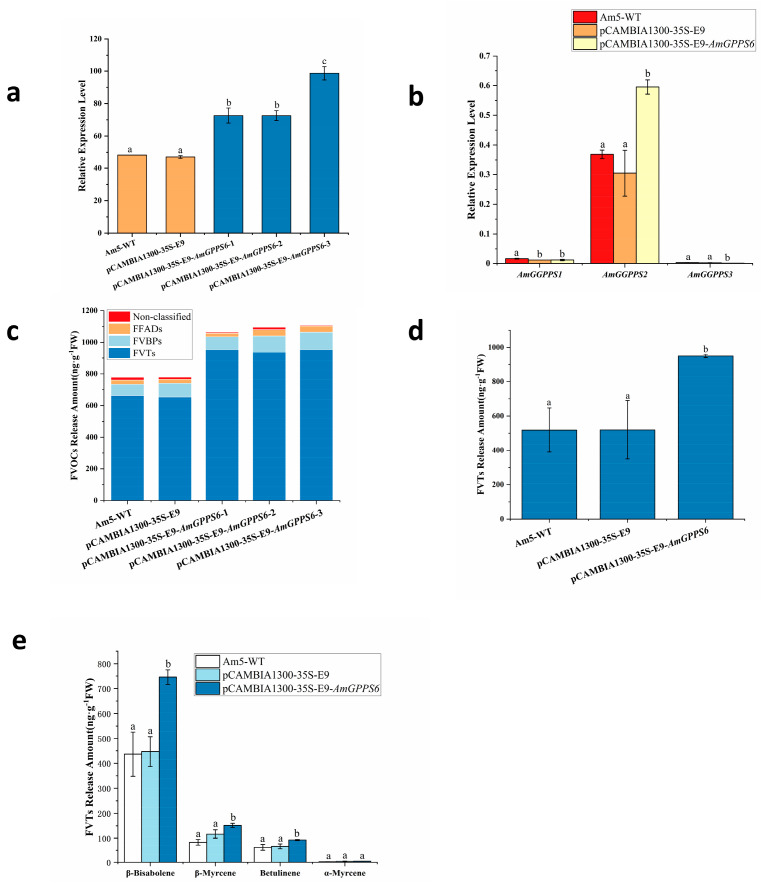
Analysis of transient overexpression of *AmGPPS6* in the homologous system of *A. majus*. (**a**) qRT-PCR analysis of *AmGPPS6* transient overexpression in *A. majus* petals; (**b**) Expression changes of *AmGGPPS* genes (*AmGGPPS1*, *AmGGPPS2*, *AmGGPPS3*) in *AmGPPS6* transiently overexpressed plants; (**c**) Effect of *AmGPPS6* transient overexpression on the total release amount of each component of floral volatile organic compounds (FVOCs) in *A. majus*, including unclassified compounds, FFADs, FVBPs, and FVTs; (**d**) Effect of *AmGPPS6* transient overexpression on the total release amount of monoterpene volatile terpenes in *A. majus*; (**e**) Effect of *AmGPPS6* transient overexpression on the release amount of main monoterpene (β-myrcene, α-myrcene) and sesquiterpenes (β-bisabolene, betulinene) components of FVTs in *A. majus*. All data are presented as mean ± SD (n = 3 biological replicates). Different lowercase letters above the bars indicate statistically significant differences between the treatment group and the control groups (Am5-WT, pCAMBIA1300-35S-E9) (*p* < 0.05, one-way ANOVA followed by Tukey’s multiple range test).

The qRT-PCR analysis (Figure 8) revealed that the relative transcript levels of *AmGPPS6* were significantly increased in plants infiltrated with pCAMBIA1300-35S-E9-*AmGPPS6* compared to both empty vector control and wild-type (WT) plants (Figure 8a), confirming successful and specific overexpression of *AmGPPS6* in the treated snapdragon plants. In these overexpression lines, transcript accumulation of *AmGGPPS1* was significantly downregulated, whereas *AmGGPPS2* exhibited highly significant upregulation, with its expression level positively correlated with that of *AmGPPS6* (Figure 8b). This coordinated expression pattern provides direct evidence at the transcriptional level supporting their potential interaction as partners in heterodimer formation, suggesting that *AmGGPPS2* likely serves as the primary interacting partner of *AmGPPS6*, directing GPP synthesis and subsequent monoterpene emission. Notably, *AmGGPPS3* transcript accumulation remained undetectable under these conditions (Figure 8b).

GC-MS analysis of floral volatile organic compounds (Figure 8c,d) demonstrated that transient overexpression of *AmGPPS6* in the weakly scented cultivar Am5 led to terpenes constituting the predominant class of floral volatiles, with total FVT emissions increased by 1.4-fold relative to controls. Specifically, compared to WT and empty vector control plants (Figure 8e), the emission level of the sesquiterpene β-bisabolene was highly significantly increased, while the emission levels of the monoterpene β-myrcene and the sesquiterpene betulinene were significantly enhanced. No significant differences were detected in the emission of other volatile compounds between overexpression lines and controls.

#### 2.5.2. Transient Silencing of the AmGPPS6 Gene in Snapdragon

**Figure 9:**
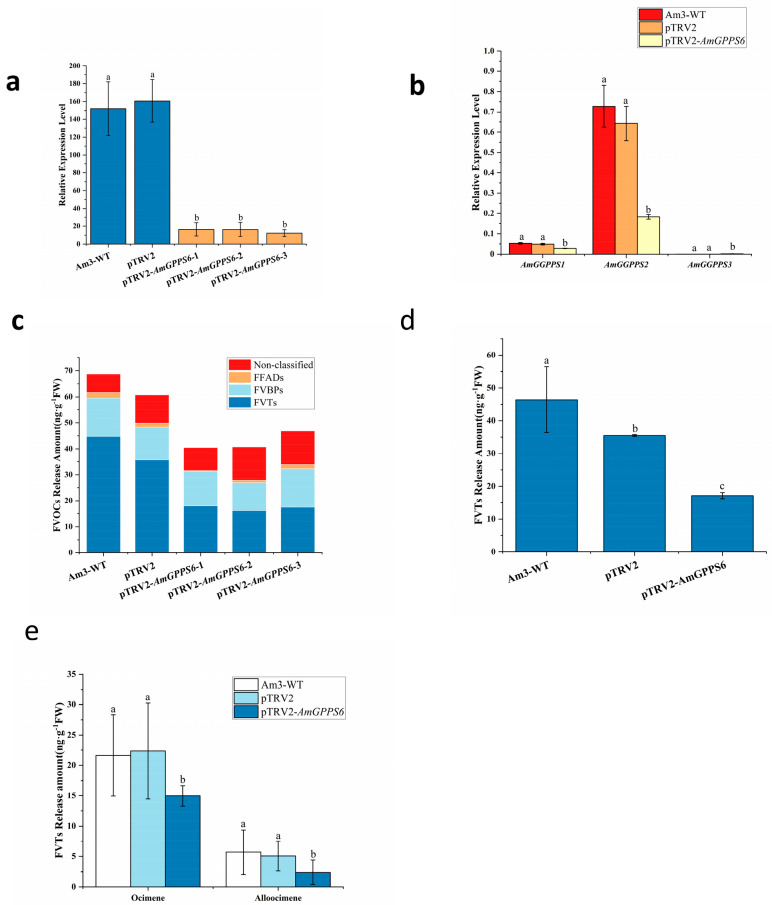
Analysis of transient silencing of *AmGPPS6* in the homologous system of *A. majus*. (**a**) qRT-PCR analysis of *AmGPPS6* transient silencing in *A. majus* petals; (**b**) Expression changes of *AmGGPPS* genes in *AmGPPS6* transiently silenced plants; (**c**) Effect of *AmGPPS6* transient silencing on the total release amount of each component of floral volatile organic compounds (FVOCs) in *A. majus*, including unclassified compounds, FFADs, FVBPs, and FVTs; (**d**) Effect of *AmGPPS6* transient silencing on the total emission of monoterpene volatile terpenes in *A. majus*; (**e**) Effect of *AmGPPS6* transient silencing on the emission of main monoterpene (β-myrcene, α-myrcene) and sesquiterpenes (β-bisabolene, betulinene) components of FVTs in *A. majus*. All data are presented as mean ± SD (n = 3 biological replicates). Different lowercase letters above the bars indicate statistically significant differences between the treatment group and the control groups (Am3-WT, pTRV2) (*p* < 0.05, one-way ANOVA followed by Tukey’s multiple range test).

The qRT-PCR analysis revealed that the relative transcript levels of *AmGPPS6* were significantly reduced in plants infiltrated with pTRV2-*AmGPPS6* compared to both empty vector control and WT plants (Figure 9a), confirming successful and specific silencing of *AmGPPS6* in the treated snapdragon plants. Upon *AmGPPS6* silencing, the expression levels of both *AmGGPPS1* and *AmGGPPS2* were significantly downregulated, consistent with the silencing trend observed for *AmGPPS6*, while *AmGGPPS3* transcripts remained virtually undetectable (Figure 9b). Notably, *AmGGPPS2*, which exhibited high basal expression levels and pronounced responsiveness to *AmGPPS6* silencing, is postulated to function as the primary interacting partner of *AmGPPS6*, orchestrating GPP synthesis and monoterpene emission.

Transient silencing of *AmGPPS6* in the strongly scented cultivar Am3 resulted in a reduced contribution of FVTs to the total FVOC profile, accompanied by a significant decrease in overall FVT emission. Specifically, the major floral scent compounds were reduced by approximately 50% compared to controls (Figure 9c,d). Among individual volatiles, the emission levels of ocimene and allo-ocimene were significantly reduced compared to those in the wild-type (WT) and empty vector control plants (Figure 9e), whereas no significant differences were detected in the emission of other volatile compounds between the treated lines and controls.

## 3. Discussion

**Figure 10:**
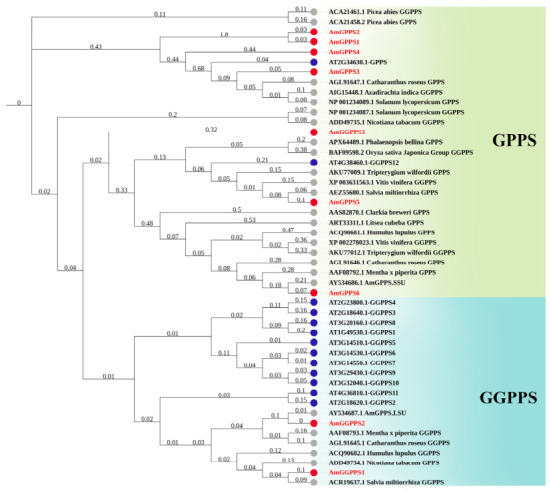
Phylogenetic tree of the GPPS/GGPPS protein. The neighbor-joining phylogenetic tree was constructed using GPPS and GGPPS protein sequences from *Antirrhinum majus* and other plant species. The tree is divided into two main clades: the GPPS clade with a green background and the GGPPS clade with a blue background. Red circles represent AmGPPS/AmGGPPS proteins from *A. majus*, blue circles represent proteins from *A. thaliana*, and gray circles represent proteins from other plant species. Branch lengths represent genetic distances, and bootstrap values are indicated at the nodes.

The *AmGPPS6* gene cloned in this study exhibits the closest phylogenetic relationship with MpGPPS.SSU from peppermint (*M. piperita*) [14,15] and shares 67.04% sequence similarity with the canonical GPPS.SSU sequence reported by Tholl et al. (2004) from the snapdragon cultivar ‘Maryland’ [13]. As both represent homologous genes from different snapdragon cultivars, they cluster together within the same clade of the phylogenetic tree (Figure 10), suggesting that *AmGPPS6* is an intra-specific orthologous evolutionary member of this canonical gene. Functional validation demonstrated that the expression level of *AmGPPS6* directly determines the inter-cultivar variation in monoterpene emission. Given that Tholl et al. (2004) did not address cultivar-specific regulation [13], we hypothesize that *AmGPPS6* may have undergone functional specialization through sequence variation during long-term cultivation and domestication, emerging as a key gene responsible for the differential floral scent among snapdragon cultivars. This provides direct evidence at the molecular evolutionary level for the diversity of floral scent traits in snapdragon.

Through bidirectional functional validation, this study reveals that the expression level of *AmGPPS6* is significantly positively correlated with monoterpene emission in snapdragon. Silencing of this gene markedly reduces the release of ocimene-class compounds, whereas its overexpression increases total volatile terpenoids by 1.4-fold, indicating that *AmGPPS6* serves as a key rate-limiting node regulating floral terpenoid biosynthetic flux. The promoter region of *AmGPPS6* contains multiple core light-responsive elements, including G-box and GT1-motif, suggesting that light signaling is a critical environmental factor regulating the expression of this gene family, and that *AmGPPS6* expression may be directly modulated by light. This finding aligns with the report by Han et al. (2022) demonstrating blue light-mediated regulation of terpene synthesis via *AmMYB24* in snapdragon [29]. We hypothesize that light may activate MYB-family transcription factors, which subsequently bind to light-responsive elements in the *AmGPPS6* promoter, modulating its expression level and consequently influencing the synthesis and emission of monoterpene floral volatiles [29].

Previous studies in transgenic tomato have demonstrated that GPP produced via the plastidial MEP pathway can be efficiently transported to the cytoplasm and preferentially utilized by cytosolic monoterpene synthases [7,30]. This directional flow of substrates across organellar boundaries represents a form of inter-compartmental metabolic channeling that prevents free substrate diffusion and metabolic competition [7,31,32]. In the present study, functional validation showed that silencing of *AmGPPS6* specifically affected the emission of ocimene and its isomers, with no significant impact on other terpenoid compounds (Figure 9c,d). In transgenic tomato, GPP produced by plastidial GPPS is exported to the cytoplasm, where it serves as a substrate for specific cytosolic monoterpene synthases [7]. Based on this, we hypothesize that the *AmGPPS6*-*AmGGPPS2* heterodimer in snapdragon may directly channel the catalytically generated GPP to downstream ocimene synthase(s), minimizing substrate diffusion and competition, thereby enabling the efficient synthesis of specific monoterpenes.

Tholl et al. (2004), based on prokaryotic heterologous expression systems, proposed the classical mechanism whereby functional GPPS requires heterodimer formation between SSU and LSU [13], without addressing the specific role or regulatory mode of endogenous LSUs in plant cells. Experimental evidence shows that *AmGPPS6* contains only two “CxxxC” auxiliary motifs and lacks the canonical “DDx_2–4_D” catalytic motif, indicating it must heterodimerize with endogenous *AmGGPPS* members possessing complete catalytic motifs to function [32,33]. In this study, overexpression of *AmGPPS6* alone significantly enhanced monoterpene synthesis, suggesting that upon binding with endogenous *AmGGPPS*, the product specificity is shifted from GGPP to GPP, thereby supporting monoterpene biosynthesis [26,33,34]. Notably, changes in *AmGPPS6* expression differentially regulate distinct *GGPPS* members: upon *AmGPPS6* silencing, both *AmGGPPS1* and *AmGGPPS2* were significantly downregulated; whereas upon overexpression, only *AmGGPPS2* was significantly upregulated, while *AmGGPPS1* was conversely downregulated. This indicates that *AmGGPPS2* is the primary functional interacting partner of *AmGPPS6*, whereas the interaction with *AmGGPPS1* may involve feedback inhibition or competitive mechanisms. This pattern differs from the SSU specifically interacting with a single LSU reported in lily [34], enabling snapdragon to flexibly utilize endogenous large subunit resources to efficiently meet monoterpene synthesis demands.

Here, overexpression and silencing of *AmGPPS6* resulted in distinct terpenoid regulatory phenotypes, with a highly significant increase in sesquiterpene emission observed upon overexpression. The significant increase in sesquiterpenes (β-bisabolene and betulinene) upon *AmGPPS6* overexpression indicates that this gene affects the overall terpenoid precursor flux rather than only monoterpene biosynthesis. This is consistent with the function of GPPS.SSU-II proteins, which can modulate the universal IPP/DMAPP pool and thereby influence both monoterpene and sesquiterpene production [33]. This may be attributed to two factors. First, the functional validation was performed using different genetic backgrounds: the weakly scented Am5 for overexpression and the strongly scented Am3 for silencing. The downstream terpene synthase (TPS) pathways may differ between these cultivars, leading to genotype-specific phenotypic outcomes that may not reflect intrinsic differences in *AmGPPS6* function itself. Second, *AmGPPS6*, as a type II small subunit (SSU-II), may, upon binding with the large subunit (LSU), not only GPP but also promote the synthesis of GGPP [35], and may possess the capacity to produce farnesyl diphosphate (FPP), thereby contributing to sesquiterpene biosynthesis. Alternatively, *AmGPPS6* may associate with bifunctional isoprenyl diphosphate synthase (IDS)/LSU to form a specialized enzyme complex [32]. Such a complex may not only catalyze GPP production but also, under specific conditions, extend carbon chain length to generate FPP (the sesquiterpene precursor) or modulate precursor flux [36], ultimately resulting in a more pronounced increase in sesquiterpenes compared to monoterpenes upon overexpression.

It should be emphasized that the inter-cultivar variation in floral scent is not determined by a single gene, but arises from the overall metabolic background differences between Am3 and Am5. The emission of monoterpenes depends on the coordinated flux of the MEP pathway [6], the activity of GPPS/GGPPS complexes, and the substrate preference of downstream terpene synthases (TPSs) [5]. In this study, *AmGPPS6* acts as the key upstream regulator that determines the precursor supply flux, while the downstream *TPS* genes specific to ocimene and other volatiles may also contribute to the final scent phenotype. Therefore, *AmGPPS6* will not be the sole determinant, but the core limiting factor that drives the differential emission between cultivars.

Although this study has elucidated the core function of *AmGPPS6* through genome-wide identification and bidirectional functional validation, certain limitations remain. First, the reliance on transient transformation systems (overexpression/VIGS) provides effects that are transient and may carry off-target risks, making it difficult to provide long-term stable functional evidence. Second, direct validation of the protein–protein interaction between *AmGPPS6* and *AmGGPPS2*, as well as functional characterization of key motifs, has not been performed. Third, the functions of other members of the *AmGPPS/GGPPS* family remain to be elucidated.

Future research should focus on three aspects. First, generation of stable genetic transformation lines for *AmGPPS6*, followed by phenotypic observation and metabolite analysis across multiple generations to further confirm the stability of gene function. Second, validation of direct interaction between *AmGPPS6* and *AmGGPPS2* using yeast two-hybrid, co-immunoprecipitation (Co-IP), and other protein–protein interaction assays, coupled with in vitro enzyme activity assays in prokaryotic systems to provide direct evidence for the catalytic mechanism. Third, functional characterization of other family members, such as *AmGPPS1/2*, to complete the regulatory network of terpenoid metabolism. The *AmGPPS6* gene identified in this study represents a key target for molecular breeding of aromatic traits in snapdragon. Future targeted modification of *AmGPPS6* holds promise for precisely developing novel snapdragon cultivars with enhanced floral scent, significantly improving their ornamental value and market competitiveness. Furthermore, this study expands the understanding of the multi-partner interaction mechanism of *AmGPPS6*, providing a new perspective for understanding the molecular evolutionary patterns underlying scent specialization in ornamental plants.

## 4. Materials and Methods

### 4.1. Experimental Materials

Inflorescences of two snapdragon cultivars Am3 and Am5, were employed for transient expression assays. Cultivar Am3 exhibits a strong floral scent, whereas Am5 displays a relatively weak scent phenotype. Plants were cultivated in a greenhouse at the Arboretum of Southwest Forestry University, Kunming, Yunnan Province, China (102.76 °E, 25.06 °N), under natural light conditions with temperatures maintained at 20–25 °C. Standard irrigation and fertilization practices were applied throughout the growth period, and plants at the flowering stage with normal development were selected for experimental use. Prior to infiltration, any open flowers were removed, and the remaining flower buds were retained for subsequent treatments. Concurrently, petal samples were collected from both Am3 and Am5 at the full flowering stage (when petals were fully expanded and floral scent emission was maximal), immediately frozen in liquid nitrogen, and stored at −80 °C for subsequent RNA extraction.

The following vectors were utilized in this study: the overexpression binary vector pCAMBIA1300-35S-E9 and VIGS vector pTRV2. The bacterial strains employed included *Escherichia coli* DH5α competent cells (Weidi Bio, Shanghai, China) and *Agrobacterium tumefaciens* GV3101 competent cells (Weidi Bio, Shanghai).

### 4.2. Identification and Physicochemical Characterization of AmGPPS/AmGGPPS Gene Family Members in Snapdragon

#### 4.2.1. Identification of Gene Family Members

The genomic sequences and gene annotation files for snapdragon were downloaded from the Snapdragon Genome Database (http://bioinfo.sibs.ac.cn/Am/, accessed on 23 March 2026). Genome assemblies, annotations, and the protein sequences of GPPS/GGPPS for *A. thaliana* were obtained from The Arabidopsis Information Resource (TAIR) database (https://www.arabidopsis.org/, accessed on 23 March 2026). Additionally, genome and annotation files for a broad range of other species—including *A. thaliana*, *Salvia miltiorrhiza*, *Nicotiana tabacum*, *Humulus lupulus*, *Catharanthus roseus*, *M*. *piperita*, *S*. *lycopersicum*, *Picea abies*, *Tripterygium wilfordii*, *Vitis vinifera*, *Litsea cubeba*, *Clarkia breweri*, *O*. *sativa* subsp. *japonica*, *Phalaenopsis bellina*, and *Azadirachta indica*—were retrieved from the National Center for Biotechnology Information (NCBI) database (https://www.ncbi.nlm.nih.gov/, accessed on 23 March 2026).

To identify putative GPPS/GGPPS homologs in snapdragon, a bidirectional BLAST search (e-value < 1 × 10^−5^) was conducted against the *A. majus* protein dataset using the *A. thaliana* GPPS/GGPPS protein sequences as queries. Concurrently, protein sequences containing the isoprenoid synthase domain (PF00348) were retrieved via searches against the Pfam database (https://pfam.xfam.org/, accessed on 23 March 2026). All candidate sequences obtained from both methods were subsequently subjected to conserved domain analysis using the NCBI Conserved Domain Database (CDD) (https://www.ncbi.nlm.nih.gov/cdd, accessed on 23 March 2026) and the SMART database (http://smart.embl-heidelberg.de/, accessed on 23 March 2026). Sequences with incomplete or truncated domains were eliminated to ensure the final set represented full-length, structurally intact GPPS/GGPPS proteins.

#### 4.2.2. Physicochemical Property Analysis and Subcellular Localization Prediction

The coding sequences (CDS) of *AmGPPS* and *AmGGPPS* genes were translated into their corresponding amino acid sequences using the Expasy Translate tool (https://web.expasy.org/translate/, accessed on 23 March 2026). Physicochemical properties of the deduced AmGPPS and AmGGPPS proteins, including molecular weight, theoretical isoelectric point (pI), and instability index, were subsequently analyzed using the Expasy ProtParam tool (https://web.expasy.org/protparam/, accessed on 23 March 2026). Subcellular localization of these proteins was predicted using the online software WoLF PSORT (https://wolfpsort.hgc.jp/, accessed on 23 March 2026).

### 4.3. Bioinformatic Analysis of the AmGPPS/AmGGPPS Gene Family in Snapdragon

#### 4.3.1. Gene Structure, Conserved Motif, and Sequence Visualization

Gene structure information for the *AmGPPS/AmGGPPS* family members was extracted from the corresponding genome annotation file (GFF format). To identify conserved protein motifs, the amino acid sequences of *AmGPPS* and *AmGGPPS* were submitted to the MEME Suite online tool (version 5.5.9; https://meme-suite.org/meme/tools/meme, accessed on 23 March 2026) [37]. The search parameters were set to discover a maximum of 15 motifs, with each motif having a width between 6 and 50 amino acids. The resulting gene structures and the distribution of conserved motifs were subsequently visualized using the “Gene Structure View” function implemented in TBtools-II (version 2.086).

#### 4.3.2. Phylogenetic Analysis and Multiple Sequence Alignment

To elucidate the evolutionary relationships, protein sequences of GPPS/GGPPS from a diverse set of species were retrieved from the National Center for Biotechnology Information (NCBI) database (https://www.ncbi.nlm.nih.gov/, accessed on 23 March 2026). The selected species included *A*. *thaliana*, *S*. *miltiorrhiza*, *N*. *tabacum*, *H*. *lupulus*, *C*. *roseus*, *M. x piperita*, *S*. *lycopersicum*, *P*. *abies*, *T*. *wilfordii*, *V*. *vinifera*, *L*. *cubeba*, *C*. *breweri*, *O*. *sativa* subsp. japonica, *P*. *bellina*, and *A*. *indica*. A phylogenetic tree was constructed using the neighbor-joining (NJ) method implemented in MEGA X software (Version 11). The Jones-Taylor-Thornton (JTT) model was selected as the amino acid substitution model. Gap handling was performed using the Partial deletion option. Nodal support was assessed via bootstrap analysis with 1000 replicates. The resulting tree was exported in Newick format and subsequently visualized and refined using the interactive Tree of Life (iTOL) online tool (https://itol.embl.de/, accessed on 23 March 2026).

For detailed sequence analysis, multiple sequence alignment of the AmGPPS and AmGGPPS proteins was performed using the Clustal W algorithm within MEGA X. The alignment was exported in FASTA format and then submitted to the ESPript 3 online server (http://espript.ibcp.fr/, accessed on 23 March 2026) for visualization of conserved domains and structural features.

#### 4.3.3. Promoter Sequence Retrieval and Cis-Element Analysis

The promoter sequences of *AmGPPS/AmGGPPS* genes were extracted from the Snapdragon Genome Database (http://bioinfo.sibs.ac.cn/Am/, accessed on 23 March 2026) using the “Gtf/Gff3 Sequences Extract” utility implemented in TBtools-II (version 2.086). Subsequently, the cis-acting regulatory elements within these promoter regions were analyzed using the online tool PlantCARE (https://bioinformatics.psb.ugent.be/webtools/plantcare/html/, accessed on 23 March 2026).

#### 4.3.4. Synteny Analysis

The syntenic relationships of *GPPS*/*GGPPS* genes among snapdragon, *A. thaliana*, tomato, rice, and maize were analyzed using the MCScanX (2012, Plant Genome Mapping Laboratory, University of Georgia, Athens, GA, USA) tool. Synteny maps were generated with TBtools to investigate the evolutionary conservation of these genes.

### 4.4. RNA Extraction and qRT-PCR

Total RNA was isolated from petals of snapdragon cultivars Am3 and Am5 at the A3 developmental stage using an RNA extraction kit. RNA purity and integrity were assessed by agarose gel electrophoresis and spectrophotometric analysis of OD260/280 ratios using a Nanodrop instrument. Purified RNA was subsequently reverse-transcribed into complementary DNA (cDNA) using the M-MLV4 First-Strand cDNA Synthesis Kit (Biomed, Beijing, China) according to the manufacturer’s instructions. Gene-specific primers for qRT-PCR analysis were designed (Table 3). The snapdragon ubiquitin gene was employed as an internal reference control. Quantitative real-time PCR was performed using the Hieff™ qPCR SYBR^®^ Green Master Mix (Yeasen, Shanghai, China) on a suitable real-time PCR detection system. The thermal cycling conditions were as follows: initial denaturation at 95 °C for 1 min, followed by 40 cycles of 95 °C for 10 s, 60 °C for 5 s, and 72 °C for 12 s. Three biological replicates were performed for each sample. Relative gene expression levels were calculated using the 2^−∆∆CT^ method.

### 4.5. Construction of Overexpression and VIGS Vectors for AmGPPS6 Functional Analysis

To investigate the biological function of *AmGPPS6*, recombinant vectors for both transient overexpression and VIGS were constructed. Full-length cDNA was synthesized from RNA extracted from snapdragon petals at the A3 developmental stage. The coding sequence of *AmGPPS6* was amplified and subsequently cloned into the overexpression binary vector pCAMBIA1300-35S-E9 using homologous recombination-based cloning.

For VIGS vector construction, optimal silencing target sites within *AmGPPS6* were predicted using an online tool. To avoid conserved domains and ensure gene-specific silencing, a fragment located outside the conserved structural regions was selected. The targeting sequence of the resulting silencing construct pTRV2-*AmGPPS6* is as follows: GGAGCAATTTTAGGTGGATGTGATGAAGATAAAATAGAAAAGTTGAGAAGATTCGGACTTTATGTGGGTACGGTTCAAGGGTTGTTGGGTAAAAATAGATCCGGATTTGAAGGAAGAATTAAGGAATTGAAGGAATTGGCTGTTAAGGAACTGGAGAGCTTTGGTGGTGAGAAAATTGAGCTGATTAGGGGCGTTTTTGAGCTAGAGCATAGTCTTGCTGGTGTTTAATTAAACACCAGCAAGACTAT. The corresponding fragment was amplified and inserted into the pTRV2 vector via homologous recombination. Both recombinant plasmids—pCAMBIA1300-35S-E9-*AmGPPS6* and pTRV2-*AmGPPS6*—along with their respective empty vector controls (pCAMBIA1300-35S-E9 empty and pTRV2 empty), were separately transformed into *Agrobacterium tumefaciens* strain GV3101. The resulting transformants harboring the empty vectors were designated to serve as negative controls for the subsequent overexpression and silencing experiments, respectively.

### 4.6. Agrobacterium-Mediated Transient Transformation of Snapdragon Inflorescences via Vacuum Infiltration

For transient transformation experiments, inflorescences of snapdragon cultivars Am3 and Am5 were selected, and all open flowers were removed, retaining only immature flower buds for subsequent infiltration. Transient transformation was performed using a modified vacuum infiltration method [38]. Agrobacterium tumefaciens strain GV3101 cultures harboring the recombinant vectors or empty vector controls were harvested by centrifugation and resuspended in infiltration medium (MS basal salt mixture 4.44 g·L^−1^, sucrose 20 g·L^−1^) to a final optical density at 600 nm (OD_600_) of 0.8. Snapdragon inflorescences were submerged in the bacterial suspension and subjected to vacuum infiltration at 0.06 MPa for 5 min. Following infiltration, the treated inflorescences were incubated in the dark at room temperature for 24 h, then transferred to a preservative solution and maintained under standard conditions.

At 7 days post-infiltration, fully opened flowers at the anthesis stage were collected. Each flower was split longitudinally into two halves: one half was used for floral volatile collection via static headspace adsorption, followed by component identification and quantification using gas chromatography–mass spectrometry (GC-MS); the other half was immediately frozen for RNA extraction and subsequent qRT-PCR analysis to assess gene expression levels.

### 4.7. GC-MS Analysis of Floral Volatile Compounds

Floral volatile organic compounds (FVOCs) were analyzed using a gas chromatography-mass spectrometry system (GCMS-QP2010SE, Shimadzu, Kyoto, Japan). The volatiles were extracted via solid-phase microextraction (SPME) using a DVB/CAR/PDMS fiber (50/30 µm, StableFlex, 1 cm length, needle size 24 ga, manual holder, plain gray hub; pk of 3). The chromatographic separation was performed using high-purity helium (99.99%) as the carrier gas at a constant flow rate of 1.0 mL·min^−1^. The injector temperature was maintained at 250 °C. The temperature program was optimized as follows: initial temperature 60 °C; increased to 70 °C at 4 °C·min^−1^ (held for 0 min); then to 74 °C at 1 °C·min^−1^ (held for 1 min); subsequently to 76.5 °C at 1 °C·min^−1^ (held for 5 min); followed by an increase to 86 °C at 1 °C·min^−1^ (held for 0 min); then to 120 °C at 2 °C·min^−1^ (held for 3 min); and finally to 180 °C at 5 °C·min^−1^ (held for 0 min). The total run time was 56.5 min.

Mass spectrometric analysis was conducted using electron impact (EI) ionization at 70 eV. The ion source temperature was set to 230 °C, and the mass scan range was *m*/*z* 29–600. The split ratio was 10:1.

The relative emission levels of FVOCs were quantified based on the peak areas of the ion chromatograms. The percentage contribution of individual volatile compounds to the total volatile profile was calculated using the normalization method. For absolute quantification, ocimene was employed as an external standard. For absolute quantification, ocimene and linalool were employed as external standards, and no internal standard was used in this study. A standard curve was constructed using the Allometric 1 model. All experimental data were converted to units of ng·g^−1^ fresh weight (FW) prior to comparative analysis and statistical evaluation. GC-MS analysis, three biological replicates were set for each sample.

## 5. Conclusions

In this study, nine *AmGPPS/AmGGPPS* family members were systematically identified at the genome-wide level in two snapdragon cultivars with contrasting floral scent intensities: the strongly scented Am3 and the weakly scented Am5. Phylogenetic analysis classified these into six *AmGPPS* and three *AmGGPPS* subfamily members. Their chromosomal locations, gene structures, protein conserved motifs, and promoter cis-acting elements were characterized, providing the first systematic overview of this gene family in snapdragon.

Comparative expression analysis during the full flowering stage revealed that *AmGPPS6* exhibited the most pronounced differential expression between cultivars, with its transcript levels showing a significant positive correlation with monoterpene emission. Bidirectional functional validation using in planta transient overexpression and VIGS demonstrated that *AmGPPS6* is a key regulator of monoterpene biosynthesis in snapdragon. Overexpression of *AmGPPS6* increased total FVTs emissions, including both monoterpenes and sesquiterpenes, whereas silencing significantly reduced the release of major floral scent compounds, specifically ocimene and its isomers, directly establishing *AmGPPS6* as the determinant of inter-cultivar scent variation. Mechanistic analysis revealed that *AmGPPS6* shares 67.04% sequence similarity with the canonical GPPS.SSU and represents an intra-specific orthologous evolutionary member. *AmGPPS6* contains only two “CxxxC” auxiliary motifs and lacks the canonical “DDx_2–4_D” catalytic motif, necessitating heterodimer formation with endogenous *AmGGPPS2*—which possesses complete catalytic motifs—to function. The strict expression coordination observed between *AmGPPS6* and *AmGGPPS2* suggests that *AmGGPPS2* serves as its primary interacting partner. Furthermore, the *AmGPPS6* promoter contains multiple core light-responsive elements, implying light-mediated regulation of its expression. Notably, functional manipulation of *AmGPPS6* specifically affected ocimene-class compounds, indicative of fine-tuned metabolic channeling.

This study unveils a molecular mechanism by which *AmGPPS6* potentially regulates monoterpene synthesis through a “flexible multi-partner interaction” mode, establishing its central role in governing cultivar-specific floral scent variation in snapdragon. These findings not only enrich the theoretical framework of plant terpenoid biosynthesis regulation but also provide a key target and technical foundation for molecular breeding of aromatic traits in snapdragon, laying a solid groundwork for developing strongly scented cultivars through gene editing and marker-assisted selection strategies.

## Figures and Tables

**Table 1:** Analysis of the Physicochemical Properties of the *Am*GPPS/*Am*GGPPS Protein in Snapdragon.

Gene ID	Gene Rename	Number of Amino Acid (aa)	Molecular Weight (kDa)	Theoretical pI	Instability Index	Aliphatic Index	GRAVY	Subcellular Localization
*Am02g09640.T01*	*AmGPPS1*	349	40.17	5.59	33.46	92.98	−0.30	CP
*Am02g45960.T01*	*AmGPPS2*	349	40.21	5.48	33.76	96.36	−0.272	CP
*Am02g01940.T01*	*AmGPPS3*	416	46.30	7.73	33.06	99.42	−0.112	CP
*Am03g05290.T01*	*AmGPPS4*	414	45.71	5.63	43.42	97.58	−0.059	CP
*Am05g42810.T01*	*AmGPPS5*	324	35.33	5.48	33.94	89.14	−0.088	CP
*Am07g02090.T01*	*AmGPPS6*	359	38.73	6.17	32.30	98.69	−0.027	CP
*Am05g01670.T01*	*AmGGPPS1*	372	40.55	6.81	39.99	95.16	−0.129	CP
*Am06g31940.T01*	*AmGGPPS2*	361	39.76	6.61	47.57	89.72	−0.135	CP
*Am06g31950.T01*	*AmGGPPS3*	297	32.43	6.27	48.61	92.36	−0.134	CP

Note: The table summarizes the physicochemical characteristics of AmGPPS/AmGGPPS proteins, including gene ID, renamed gene name, amino acid number (aa), molecular weight (kDa), theoretical isoelectric point (pI), instability index, aliphatic index, grand average of hydropathicity (GRAVY), and subcellular localization. CP, chloroplast.

**Table 2:** Inter-species collinearity analysis of *AmGPPS*/*AmGGPPS* homologous genes.

Snapdragon *AmGPPS*/*GGPPS* Gene	Collinearity Analysis of Homologous Genes Among Species	Origin of Species
*Am05g42810.T01*	*NC_089036.1*	*S*. *lycopersicum*
*Am02g01940.T01*	*AT2G34630.2*	*A. thaliana*
*Am02g09640.T01*	*AT4G17190.1*	
*Am02g09640.T01*	*AT5G47770.1*	
*Am03g05290.T01*	*AT1G17050.1*	
*Am05g42810.T01*	*AT4G38460.1*	
*Am05g01670.T01*	*AT4G36810.1*	
*Am06g31940.T01*	*AT2G18620.1*	
*Am06g31940.T01*	*AT4G36810.1*	
*Am05g01670.T01*	*NC_015439.3*	*O*. *sativa*
*Am05g01670.T01*	*NC_015441.3*	
*Am06g31940.T01*	*NC_015439.3*	
*Am07g02090.T01*	*NC_015444.3*	

Note: The table lists the collinear orthologous gene pairs of *AmGPPS/AmGGPPS* genes between snapdragon (*A. majus*) and other plant species, including *S. lycopersicum*, *A. thaliana*, and *O. sativa*.

**Table 3:** qRT-PCR Primer Sequences.

Gene	Primer Sequence (5′-3′)	
q*AmUBI*	F: AGCCGATGGAAGTATATGTTTGGACATC	R: CTAACTTTGCGGTTATAATCTCGTTTA
q*AmGPPS1*	F: ACTGCTTTGGTGAACCGGAA	R: TTGCAACGTCAGCAGGATCT
q*AmGPPS2*	F: TGCCACTTCACCGACGTATC	R: CAACCAGCCACGAACACTTG
q*AmGPPS3*	F: TTCCTTGTCAGACATCCGCC	R: TGCAAGCTCCCTAGTCCTCT
q*AmGPPS4*	F: AAGAGGAAAGGCAACCGTCC	R: CAACGTCGCAGTCGAACAAG
q*AmGPPS5*	F: CCGAACCAGATCTACGAGGC	R: ACAATGCGTCTCCGGCTAAA
q*AmGPPS6*	F: ATCCGTATTCGAGCCCATGC	R: CCATCAACTCCAGCCCGAAT
q*AmGGPPS1*	F: TTGTTGGAAGCGTCTGTGGT	R: TTCTCCGCAAACTCCCTAGC
q*AmGGPPS2*	F: TTTACGACGTGGAAAGCCCA	R: AAAACCACCGAAGCCTCCAA
q*AmGGPPS3*	F: CGACGATTTGCCGTGTATGG	R: CCCCTCTGGTCCAATCAACC

Note: The table lists the forward (F) and reverse (R) primer sequences (5′ to 3′) used for qRT-PCR expression analysis of *AmGPPS/AmGGPPS* genes, with *AmUBI* as the reference gene.

## Data Availability

The original contributions presented in this study are included in the article/Supplementary Material. Further inquiries can be directed to the corresponding authors.

## Supplementary Materials

The following supporting information can be downloaded at: https://www.mdpi.com/article/10.3390/plants15101457/s1.
